# Editorial: Epigenetic and genetic mechanisms underlying cardiovascular diseases and neurodevelopmental disorders, volume II

**DOI:** 10.3389/fmolb.2026.1896846

**Published:** 2026-06-19

**Authors:** Peng Zhang, Lingshan Gou, Chang-Bo Zheng

**Affiliations:** 1 Department of Otolaryngology, Shenzhen Longgang Otolaryngology Hospital and Shenzhen Institute of Otolaryngology, Shenzhen, Guangdong, China; 2 Center for Genetic Medicine, Xuzhou Maternity and Child Healthcare Hospital Affiliated to Xuzhou Medical University, Xuzhou, Jiangsu, China; 3 School of Pharmaceutical Science and Yunnan Key Laboratory of Pharmacology for Natural Products, Kunming Medical University, Kunming, Yunnan, China

**Keywords:** cardiac malformations, cardiovascular diseases, epigenetic, etiology, genetic

As major threats to human health and quality of life, cardiovascular diseases (CVD) and neurodevelopmental disorders remain critical global health challenges. The intricate pathogenesis of these conditions, which arises from multifactorial etiologies integrating genetic variants, epigenetic alterations, and environmental exposures, has been extensively elucidated in previous studies (Hartiala et al., 2021; Tada et al., 2022; Shi et al., 2022). Dissecting these etiological mechanisms is pivotal to advancing targeted therapeutic strategies for clinical management. This Research Topic presents ten articles including eight original research and two review articles, that collectively explore the genetics and epigenetic bases, as well as therapeutic targets, underlying these conditions.

Epigenetic regulation, encompassing mechanisms such as DNA methylation, histone modification, and non-coding RNAs, serves as a critical intermediary, enabling environmental factors to influence gene expression without altering the DNA sequence itself, and plays a pivotal role in the onset and progression of CVD (Hartiala et al., 2021; Shi et al., 2022; Pang et al., 2025; Gao et al., 2026). Gao et al. conducted a comprehensive review of the epigenetic mechanisms involved in coronary artery disease, integrating evidence on DNA methylation, histone modifications, and non-coding RNAs that drive disease progression. The review underscored how these reversible modifications respond to environmental risk factors and contribute to pathological processes, thereby presenting potential therapeutic targets. Additionally, the review summarized epigenetic biomarkers relevant to the diagnosis and prognosis of coronary artery disease. In addition to this review, He et al. utilized integrated expression quantitative trait loci and Mendelian randomization analyses to identify 13 candidate genes associated with myocardial infarction, such as ADM, BCL6, and BNIP3L, which are involved in mitochondrial apoptotic and mitochondrial organization regulatory pathways. Their research elucidated candidate genes and biological processes implicated in the pathogenesis of myocardial infarction.

Kawasaki disease (KD), an acute vasculitis primarily affecting young children, presents a significant challenge in pediatric healthcare. Nonetheless, the cellular heterogeneity and underlying pathogenic mechanisms of this disease remain inadequately understood (Vaňková et al., 2023; Burns, 2024). Three original research papers in this Research Topic concentrated on the pathogenic mechanisms, molecular mechanisms of resistance to intravenous immunoglobulin and aspirin, and diagnostic biomarkers underlying KD. Through proteomic analysis, Fan et al. identified 12 differentially expressed proteins in gingival crevicular fluid, such as SAA1, IFIT3, and MX1, as potential non-invasive biomarkers. By integrating single-cell RNA sequencing with single-cell assay for transposase-accessible chromatin sequencing, Fan et al. further conducted a detailed analysis of immune cell heterogeneity in KD. Their study identified dysregulated pathways in T cells, B cells, and NK cells, and pinpointed specific subsets associated with resistance to intravenous immunoglobulin treatment. Building on this research, Jiang et al. explored the hematologic dynamics and genetic factors contributing to aspirin resistance in KD. Their longitudinal study revealed phase-dependent changes in platelet parameters and identified the rs8090438 variant in the MBP gene as a potential genetic factor associated with reduced aspirin responsiveness.

Pulmonary arterial hypertension and chronic heart failure present substantial challenges within the domain of CVD (Chizinga and Fares, 2018; Heidenreich et al., 2022; Wu et al., 2025; Liu et al., 2026). In this Research Topic, one original research paper and one in-depth review paper concentrated on the pathogenic mechanisms and therapeutic targets underlying pulmonary arterial hypertension. Liu et al. conducted a study utilizing integrated transcriptomic, proteomic, and phosphoproteomic analyses, which identified dysregulated pathways in autophagy, apoptosis, and HIF-1 signaling. The study provided a molecular framework for comprehending the mechanisms underlying pulmonary vascular remodeling. They further validated alterations in the expression of enolase 1, chloride intracellular channel 1, caveolin-2, and eukaryotic translation initiation factor 2A, thereby highlighting potential therapeutic targets. Concurrently, Li et al. reviewed emerging pharmacological interventions targeting genetic variants associated with chronic heart failure, including SGLT2 inhibitors, angiotensin receptor neprilysin inhibitors, vericiguat, levosimendan, and ivabradine. The review elucidated their mechanisms of action, such as modulation of the AMPK, NHE1, and NPR3 pathways. Furthermore, the review emphasized the significance of personalized medicine guided by genetic polymorphisms, the discovery of novel targets, and the utilization of breakthrough technologies, including artificial intelligence-assisted decision-making and gene editing to further improve the outcomes in chronic heart failure.

Abnormalities in the genetic architecture of CVD have been extensively documented; however, their genetic etiologies remain largely elusive (Heimlich and Bick, 2022; Yu et al., 2023; Qiao et al., 2025). Addressing this critical knowledge gap, two original research papers featured in this Research Topic delve into the underlying genetic mechanisms driving these conditions. Lu et al. identified a novel frameshift mutation, c.2517_2518insCA, in the LDLR gene within a Chinese family affected by familial hypercholesterolemia (FH), thereby enhancing the understanding of phenotype-genotype correlations associated with LDLR mutations in FH. The proband achieved effective lipid control and a favorable prognosis through personalized pharmacotherapy, including statins, ezetimibe, and PCSK9 inhibitors. Their study highlights the critical importance of genetic testing in the diagnosis and clinical management of FH. Furthermore, Wang et al. demonstrated that fetuses with cardiac malformations accompanied by structural extracardiac anomalies exhibit the highest incidence of chromosomal aberrations, compared to those with simple or complex cardiac malformations, or cardiac malformations combined with soft markers. Their study advocated that the integration of karyotyping and chromosomal microarray analysis be considered the preferred approach for comprehensive cytogenetic evaluation, whereas isolated simple malformations or those accompanied by soft markers might warrant case-by-case evaluation, allowing for tailored diagnostic strategies based on clinical context.

Furthermore, the study by Zu et al. explored sex-specific alterations in the salivary microbiome of patients with Parkinson’s disease. Utilizing 16S rRNA sequencing, they identified distinct taxonomic patterns: female patients exhibited an increase in Prevotella and Veillonella but a decrease in Akkermansia, while male patients displayed a TM7-skewed profile characterized by an abundance of Candidatus Saccharimonas and a reduction in *Haemophilus*. These sex-specific microbial changes correlated with the severity of both motor and non-motor symptoms, suggesting that salivary biomarkers could offer significant potential for enhancing precision medicine approaches.

In conclusion, this Research Topic provides a comprehensive and compelling update on the rapidly evolving landscape of genetic and epigenetic mechanisms underlying cardiovascular and neurological diseases. The findings not only bridge the gap between basic molecular research and therapeutic applications but also offere a robust foundation for developing targeted interventions and personalized management strategies, advancing the shared goal of translating genomic and epigenomic insights into tangible improvements in patient care.
